# Aspects of acute enterocolitis with rotavirus at children hospitalized in the Clinical Hospital of Infectious Diseases Constanța in 2011-2012

**DOI:** 10.1186/1471-2334-13-S1-P105

**Published:** 2013-12-16

**Authors:** Simona Diaconu, Sorin Rugină

**Affiliations:** 1 Clinical Hospital of Infectious Diseases, Constanța, Romania; 2 Ovidius University, Constanța, Romania

## Background

Gastroenteritis with rotavirus is the leading cause of severe diarrhea among infants and young children. It is transmitted by fecal-oral route, via contact with contaminated hands, surfaces and objects, and possibly by the respiratory route.

We aimed to evaluate the incidence of nosocomial infections with rotavirus in children, along with the clinical and therapeutic aspects of the disease.

## Methods

We conducted an observational, retrospective study that included children hospitalized in the Clinical Hospital of Infectious Diseases Constanța in 2011-2012.

We analyzed the medical records of the patients and extracted demographic data, temperature, frequency of vomiting and diarrheic stools, degree of dehydration, duration of parenteral rehydration and hospitalization.

We used a rapid latex agglutination assay for the detection of rotavirus in fecal samples.

## Results

From the total of 505 patients with ages between 4 months and 12 years diagnosed with enterocolitis with rotavirus, 45 were cases of nosocomial infection, from pediatric general ward, pediatric surgery, infectious diseases, intensive care unit.

The incidence was increased during winter and spring. Sex-ratio M:F=1.2:1. Children between 1-3 years were more affected than the others (63.60%). Rotaviral gastroenteritis is a mild to severe disease characterized by vomiting, watery diarrhea, fever. Dehydration is more common in rotaviral infection than in most of those caused by bacterial pathogens. Treatment is nonspecific and involves maintenance of hydration.

## Conclusion

Gastroenteritis with rotavirus is one of the most important causes of nosocomial diarrhea in children and can prolong the period of hospitalization. The contagiosity is very high.

